# Comparison of adjuvant nab-paclitaxel plus gemcitabine, S-1 and gemcitabine chemotherapy for resectable pancreatic cancer: a real-world study

**DOI:** 10.3389/fonc.2023.1276037

**Published:** 2023-10-16

**Authors:** Haorui Li, Yu Guo, Xugang Sun, Yang Lu, Shaofei Chang, Xiuchao Wang, Song Gao, Chuntao Gao, Tiansuo Zhao

**Affiliations:** ^1^ Department of Pancreatic Cancer, Tianjin Medical University Cancer Institute and Hospital, National Clinical Research Center for Cancer, Tianjin’s Clinical Research Center for Cancer, Tianjin, China; ^2^ Department of Gastrointestinal Pancreatic Surgery, Shanxi Provincial People’s Hospital, Taiyuan, China

**Keywords:** adjuvant chemotherapy, resectable pancreatic cancer, real-world study, overall survival, recurrence-free survival

## Abstract

**Background:**

A survival benefit has been seen for both adjuvant nab-paclitaxel plus gemcitabine (AG) and S-1 chemotherapy compared to gemcitabine (GEM) for resectable pancreatic cancer in the APACT (2019) and JASPAC01 trials (2016), respectively. However, supporting evidence regarding the effectiveness of AG or S-1 compared to gemcitabine in real-world clinical practice remains lacking.

**Methods:**

Our study included all 246 pancreatic cancer patients who underwent surgical treatment and received postoperative adjuvant chemotherapy with AG, S-1, or GEM except for those meeting exclusion criteria (R2 resection, neoadjuvant therapy, or synchronous malignancy) at Tianjin Medical University Cancer Institute and Hospital from June 2015 to July 2021. The primary outcome was overall survival (OS) and recurrence-free survival (RFS).

**Results:**

In total, 246 patients were included, of whom 54(22%) received adjuvant AG, 103(41%) received adjuvant S-1, and 89(37%) received adjuvant GEM. Adjuvant S-1 was associated with a prolonged OS compared to GEM (median OS S-1 vs GEM: 27.0 vs 20.0 months; HR: 0.65, P = .016) and a significantly prolonged RFS compared to GEM (median RFS S-1 vs GEM: 20.0 vs 8.2 months; HR: 0.58, P = .002). After adjusting for known prognostic factors in multivariate Cox regression analysis, this survival benefit persists and is consistent in most subgroups in our subgroup analysis. However, no statistically significant differences in OS or RFS were seen between patients treated with AG and patients treated with GEM.

**Conclusions:**

In this retrospective real-world study, adjuvant S-1 chemotherapy was associated with improved survival compared to GEM while no differences in OS or RFS were observed for AG compared to GEM.

## 1 Introduction

Pancreatic ductal adenocarcinoma (PDAC) is highly malignant and has become a common cause of cancer-related death in humans worldwide (1, 2). It has been reported that the 5-year overall survival (OS) is only about 11% (3, 4). At the time of diagnosis, most patients present with locally advanced or metastatic disease, and only one-fifth of the patients are able to undergo surgical resection, which poses a great challenge for the treatment of the disease (5). Although surgical treatment is still the preferred treatment for pancreatic cancer, surgical resection alone is not sufficient to overcome the risk of local or distant recurrence in most patients with PDAC (6, 7).

Since the late 20th into the early 21st century, growing evidence has indicated that oncologic outcomes including overall survival and time to recurrence can be improved by treatment with adjuvant chemotherapy following surgical resection for pancreatic cancer (8). Adjuvant therapy in Western countries and patients who undergo radical surgery are typically treated with adjuvant chemotherapy if there is no contraindication (7, 9, 10). However, in recent years, several clinical trials have confirmed that compared with traditional gemcitabine monotherapy, fluorouracil-based or gemcitabine-based combination chemotherapy schemes contribute to prolonging the postoperative disease-free survival and overall survival of patients with pancreatic cancer (11–15).

In 2019, the APACT study (an international multicenter phase III randomized controlled clinical trial) compared nab-paclitaxel combined with gemcitabine (AG) scheme with GEM alone (16). The median OS for patients treated with AG was 41.8 months compared to 37.7 months for patients treated with GEM (hazard ratio [HR]: 0.82, 95% confidence interval [CI] 0.687-0.973, P = .0232) with an acceptable level of treatment-related adverse events, which suggests AG scheme may be valuable in improving the prognosis of resectable pancreatic cancer patients. In 2016, a clinical trial conducted in Japan showed that S-1 (a combination of tegafur, gimeracil, and interacial potassium) was superior to gemcitabine as adjuvant therapy, with more than 40% 5-year survival for the S-1 group versus 24% in the gemcitabine group, which suggests S-1 may be used as an alternative to adjuvant chemotherapy for resectable PDAC (12).

However, current evidence on the survival advantage of the AG or S-1 scheme to GEM monotherapy for PDAC is still limited to clinical trials. Since clinical trial results are not always repeatable in the real world, our study aims to evaluate whether AG or S-1 scheme can improve the clinical prognosis of patients with resectable pancreatic cancer compared with adjuvant GEM in our cohort in real-world clinical practices.

## 2 Patients and method

### 2.1 Study population

This retrospective study included 246 patients who underwent surgical treatment with a postoperative pathological diagnosis of pancreatic cancer at Tianjin Medical University Cancer Institute and Hospital from June 2015 to July 2021. Additional inclusion criteria were undergoing postoperative chemotherapy treatment with adjuvant GEM monotherapy, adjuvant AG, or adjuvant S-1. All patients who received at least one cycle of postoperative adjuvant chemotherapy were included. Exclusion criteria were a resection with macroscopic residual tumor (R2), synchronous malignancy of another organ, with neoadjuvant chemotherapy before the surgery, without postoperative adjuvant chemotherapy, or with postoperative adjuvant chemotherapy other than GEM, AG, or S-1 scheme.

### 2.2 Data collection

Information on patient and tumor characteristics, treatment, and clinical outcomes is routinely extracted from the medical records according to standardized definitions. Patient characteristics included sex, age, American Society of Anesthesiologists (ASA) score, and CA19-9 value before the operation. Tumor characteristics included the tumor location, pathological tumor size, number of positive lymph nodes, tumor differentiation grade, and TNM classification. For our study, the TNM stage classification was converted to the 8th edition of the American Joint Committee on Cancer for all patients, mainly using the number of positive lymph nodes and pathological tumor size (17). The main clinical outcomes included OS (overall survival), measured from the start of adjuvant chemotherapy until death from any cause, and RFS (recurrence-free survival), calculated as the interval between the date of surgery and the first date of imaging evidence of tumor recurrence. Patients alive or without recurrence at the last follow-up were censored. Follow-up was completed until 1 June 2022. Detailed follow-up data were available for approximately 85% of patients.

### 2.3 Statistical analysis

The Multiple interpolation method is used to interpolate the missing data. If the quantitative data meet the normal distribution, using mean ± standard deviation to express the data, and comparison between groups using the single factor analysis of variance and Student’ *t*-tests; however, if the normal distribution is not satisfied, the median and interquartile range (IQR) are used, and the non-parametric Wilcoxon signed-rank test is used for comparison between groups. Categorical variables were summarized as numbers and percentages and compared using the Chi-square test or Fisher’s exact test. OS and RFS were estimated according to the Kaplan-Meier method and the difference in survival among the three treatment groups was analyzed using the log-rank test. In addition, univariable and multivariable Cox regression analyses were performed to assess the treatment effect expressed as HR with corresponding 95% CI. Furthermore, the treatment effect among the three treatment groups was assessed in prespecified subgroups according to the Cox regression model with subgroups based on age, sex, CA19-9, tumor location, TNM stage, pathological tumor size, lymph nodes, and tumor differentiation. All tests were two-sided and the difference was considered statistically significant at P<0.05 bilaterally. All analyses were performed using the SPSS 26.0 statistical software and GraphPad Prism 8.0.

## 3 Results

### 3.1 Comparison of the baseline characteristics

**Figure 1:**
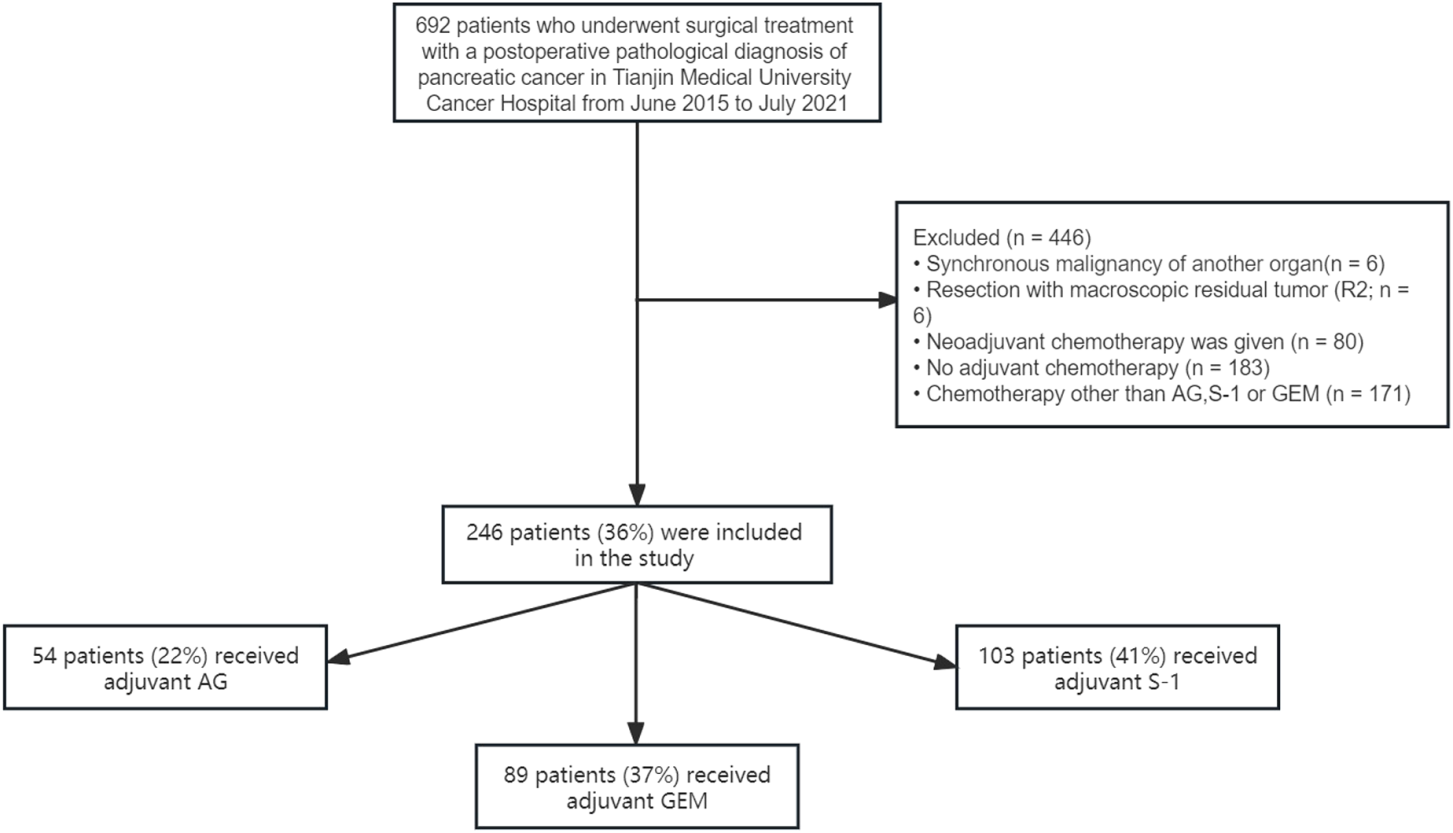
Selection of the study population. Selection of the study population. GEM, gemcitabine monotherapy; AG, gemcitabine combined with nab-paclitaxel; S-1, S-1 monotherapy.

Our database contained data on 692 patients who underwent surgical treatment with a postoperative pathological diagnosis of pancreatic ductal adenocarcinoma (PDAC) at Tianjin Medical University Cancer Institute and Hospital from June 2015 to July 2021. After applying the specific eligibility criteria, 246 patients were included, of whom 54 (22%) received adjuvant AG chemotherapy, 103 (41%) received adjuvant S-1 chemotherapy, and 89 (37%) received adjuvant GEM chemotherapy (**Figure 1**). As for these 246 patients, 61% of the patients were male, the median age was 59.22 ± 8.07 years and 58.1% of the patients had CA19-9 value<300 before the operation. Besides, according to postoperative pathological results, most patients were diagnosed at TNM stage I (47.1%), followed by stage II (37.0%), and stage III (15.9%). 76.8% of the patients presented with no tumor metastasis in lymph nodes and 53.7% of the patients presented with pathological tumor size <30mm. No statistically significant differences in characteristics were seen among the three treatment groups (**Table 1**).

**Table 1:** Baseline characteristics.

Number of patients	Overall 246	AG 54	S-1 103	Gem 89	P value
**Age,years(mean ± SD)**	59.22 ± 8.07	58.96 ± 8.53	58.37 ± 7.82	60.37 ± 8.02	0.223
**Sex, n (%)**					0.942
Male	150(61.0)	34(63.0)	62(60.2)	54(60.7)	
Female	96(39.0)	20(37.0)	41(39.8)	35(39.3)	
**ASA score, n (%)**					0.992
I	40(16.2)	10(17.7)	16(15.3)	14(16.2)	
II	135(54.9)	29(52.9)	57(55.5)	49(54.8)	
III	71(28.9)	15(29.4)	30(29.2)	26(29.0)	
**CA19-9, n (%)**					0.559
<300U/mL	143(58.1)	28(51.9)	61(59.2)	54(60.7)	
≥300U/mL	103(41.9)	26(48.1)	42(40.8)	35(39.3)	
**Tumor differentiation, n (%)**					0.789
Poor/undifferentiated	134(54.5)	29(53.7)	54(52.4)	51(57.3)	
Well/moderate	112(45.5)	25(46.3)	49(47.6)	38(42.7)	
**TNM stage, n (%)**					0.174
I	116(47.1)	20(37.0)	50(48.5)	46(51.7)	
II	91(37.0)	20(37.0)	38(36.9)	33(37.1)	
III	39(15.9)	14(26.0)	13(14.6)	10(11.2)	
**Tumor location, n (%)**					0.123
Head	164(66.6)	34(63.0)	65(63.1)	65(73.0)	
Body	56(22.8)	15(27.8)	29(28.2)	12(13.5)	
Tail	26(10.6)	5(9.2)	9(8.7)	12(13.5)	
**Lymph nodes, n (%)**					0.496
Negative	189(76.8)	40(74.1)	83(80.6)	66(74.2)	
Positive	57(23.2)	14(25.9)	20(19.4)	23(25.8)	
**Pathological tumor size, n (%)**					0.936
<30mm	132(53.7)	29(53.7)	54(52.4)	49(55.0)	
≥30mm	114(46.3)	25(46.3)	49(47.6)	40(45.0)	

AG, nab-paclitaxel combined with gemcitabine; Gem, gemcitabine; ASA, American Society of Anesthesiologists; CA19-9, carbohydrate antigen 19-9; TNM stage: Tumor, Node, Metastasis stage, according to AJCC 8th edition.

### 3.2 Survival analysis

**Figure 2:**
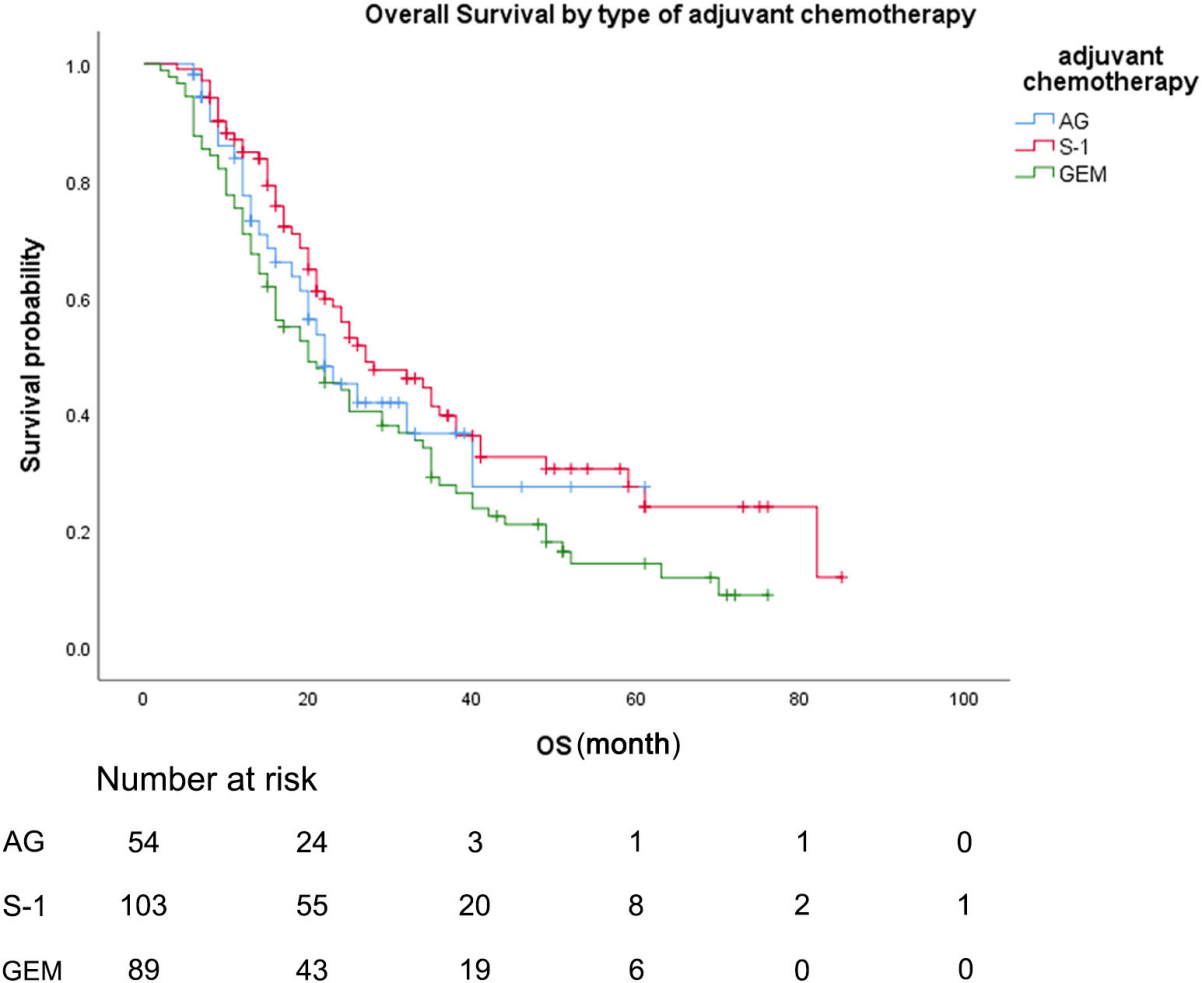
Overall survival, by type of adjuvant chemotherapy. Overall survival, by type of adjuvant chemotherapy. Kaplan-Meyer survival curves comparing overall survival, by type of adjuvant chemotherapy. GEM, gemcitabine monotherapy; AG, gemcitabine with nab-paclitaxel; S-1, S-1 monotherapy.

**Figure 3:**
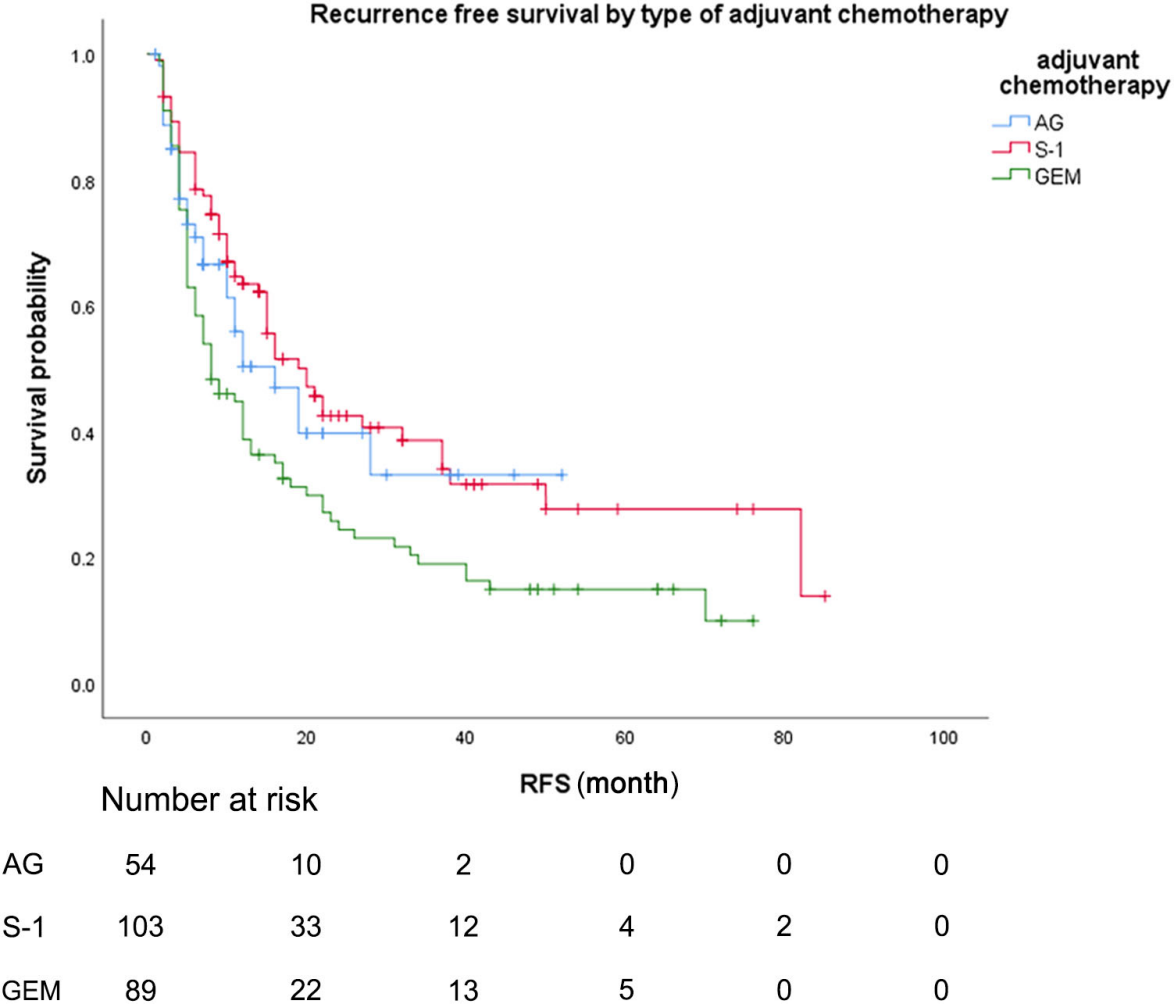
Recurrence-free survival, by type of adjuvant chemotherapy. Recurrence-free survival, by type of adjuvant chemotherapy. Kaplan-Meyer survival curves comparing recurrence-free survival, by type of adjuvant chemotherapy. GEM, gemcitabine monotherapy; AG, gemcitabine with nab-paclitaxel S-1, S-1 monotherapy.

During the follow-up period, the median follow-up time for patients alive at the last follow-up was 30.8 months. Median OS was 22.0 months (95% CI 15.7-28.3) for patients treated with AG chemotherapy, 27.0 months (95% CI 17.4-36.6) for patients treated with S-1 chemotherapy, and 20.0 months (95% CI 13.0-27.0) for patients treated with GEM chemotherapy (**Figure 2**). Median RFS was 16.0 months (95% CI 8.4-23.6) for patients treated with AG chemotherapy, 20.0 months (95% CI 14.3-25.7) for patients treated with S-1 chemotherapy, and 8.2 months (95% CI 4.2-11.8) for patients treated with GEM chemotherapy (**Figure 3**).

As for the comparison between three groups of patients, the Median OS for patients treated with S-1 was 27.0 months (95% CI 17.4-36.6) compared to 20.0 months (95% CI 13.0-27.0) for patients treated with GEM (unadjusted HR: 0.65, 95% CI 0.46-0.82, P = .016). Median RFS for patients treated with S-1 was 20.0 months (95% CI 14.3-25.7) compared to 8.2 months (95% CI 4.2-11.8) for patients treated with GEM (unadjusted HR: 0.58, 95% CI 0.41-0.82, P = .002). No statistically significant differences in OS or RFS were seen between the patients treated with AG and patients treated with GEM or between the patients treated with AG and patients treated with S-1 (median OS AG vs GEM: 22.0 vs 20.0 months; HR: 0.80, P = .336; median RFS AG vs GEM: 16.0 vs 8.2 months; HR: 0.70, P = .113).

Univariable and multivariable analyses showed that besides treatment, the value of CA19-9 before the operation, pathological tumor size, lymph node involvement, TNM stage, and tumor differentiation were all associated with OS (**Table 2**) and RFS (**Table 3**). These factors mentioned above were the independent predictors of overall survival and recurrence-free survival.

**Table 2:** Univariable and multivariable analysis of clinicopathological factors for OS.

Factors	Number of patients	Univariable analysis		Multivariable analysis	
		HR (95% CI)	P value	HR (95% CI)	P value
Age					
<60 years	131	1 [Reference]			
≥60 years	115	0.971(0.709-1.332)	0.857		
Sex					
Male	150	1 [Reference]			
Female	96	1.000(0.724-1.382)	0.998		
Treatment					
AG	54	0.804(0.515-1.255)	0.336		
S-1	103	0.652(0.461-0.922)	0.016^a^	0.561(0.394-0.800)	0.001^a^
G	89	1 [Reference]		1 [Reference]	
CA199					
<300U/mL	143	1 [Reference]		1 [Reference]	
≥300U/mL	103	1.738(1.266-2.386)	<0.001^a^	1.680(1.214-2.325)	0.002^a^
Tumor differentiation					
Poor/undifferentiated	134	1 [Reference]		1 [Reference]	
Well/moderate	112	0.614(0.446-0.844)	0.003^a^	0.664(0.479-0.920)	0.014^a^
TNM stage					
I	116	1 [Reference]		1 [Reference]	
II	91	1.094(0.772-1.550)	0.615		
III	39	3.211(2.059-5.006)	<0.001^a^	2.459(1.542-3.919)	<0.001^a^
Tumor location					
Head	164	1 [Reference]			
Body	56	1.365(0.939-1.986)	0.103		
Tail	26	0.928(0.548-1.573)	0.782		
Lymph nodes					
Negative	189	1 [Reference]		1 [Reference]	
Positive	57	2.464(1.738-3.493)	<0.001^a^	2.154(1.496-3.102)	<0.001^a^
Pathological tumor size					
<30mm	132	1 [Reference]		1 [Reference]	
≥30mm	114	1.578(1.152-2.161)	0.004^a^	1.547(1.093-2.188)	0.014^a^

CI, confidence interval; HR, hazard ratio; AG, nab-paclitaxel combined with gemcitabine; Gem, gemcitabine; CA19-9, carbohydrate antigen 19-9; TNM stage: Tumor, Node, Metastasis stage, according to AJCC 8th edition.

^a^P <.05.

**Table 3:** Univariable and multivariable analysis of clinicopathological factors for RFS.

Factors	Number of patients	Univariable analysis		Multivariable analysis	
		HR (95% CI)	P value	HR (95% CI)	P value
Age					
<60 years	131	1 [Reference]			
≥60 years	115	0.939(0.685-1.287)	0.695		
Sex					
Male	150	1 [Reference]			
Female	96	0.989(0.716-1.367)	0.948		
Treatment					
AG	54	0.699(0.449-1.089)	0.113		
S-1	103	0.581(0.411-0.822)	0.002^a^	0.500(0.351-0.713)	<0.001^a^
G	89	1 [Reference]		1 [Reference]	
CA19-9					
<300U/mL	143	1 [Reference]		1 [Reference]	
≥300U/mL	103	1.723(1.252-2.369)	<0.001^a^	1.612(1.163-2.236)	0.004^a^
Tumor differentiation					
Poor/undifferentiated	134	1 [Reference]		1 [Reference]	
Well/moderate	112	0.611(0.444-0.840)	0.002^a^	0.604(0.436-0.837)	0.002^a^
TNM stage					
I	116	1 [Reference]		1 [Reference]	
II	91	1.136(0.802-1.610)	0.472		
III	39	2.550(1.630-3.988)	<0.001^a^	1.888(1.159-3.075)	0.011^a^
Tumor location					
Head	164	1 [Reference]			
Body	56	1.339(0.921-1.947)	0.126		
Tail	26	0.933(0.551-1.581)	0.797		
Lymph nodes					
Negative	189	1 [Reference]		1 [Reference]	
Positive	57	2.519(1.770-3.585)	<0.001^a^	2.212(1.528-3.202)	<0.001^a^
Pathological tumor size					
<30mm	132	1 [Reference]		1 [Reference]	
≥30mm	114	1.663(1.215-2.278)	0.014^a^	1.623(1.151-2.288)	0.006^a^

CI, confidence interval; HR, hazard ratio; AG, nab-paclitaxel combined with gemcitabine; Gem, gemcitabine; CA19-9, carbohydrate antigen 19-9; TNM stage: Tumor, Node, Metastasis stage, according to AJCC 8th edition.

^a^P <.05.

### 3.3 Subgroup analysis of survival

**Figure 4:**
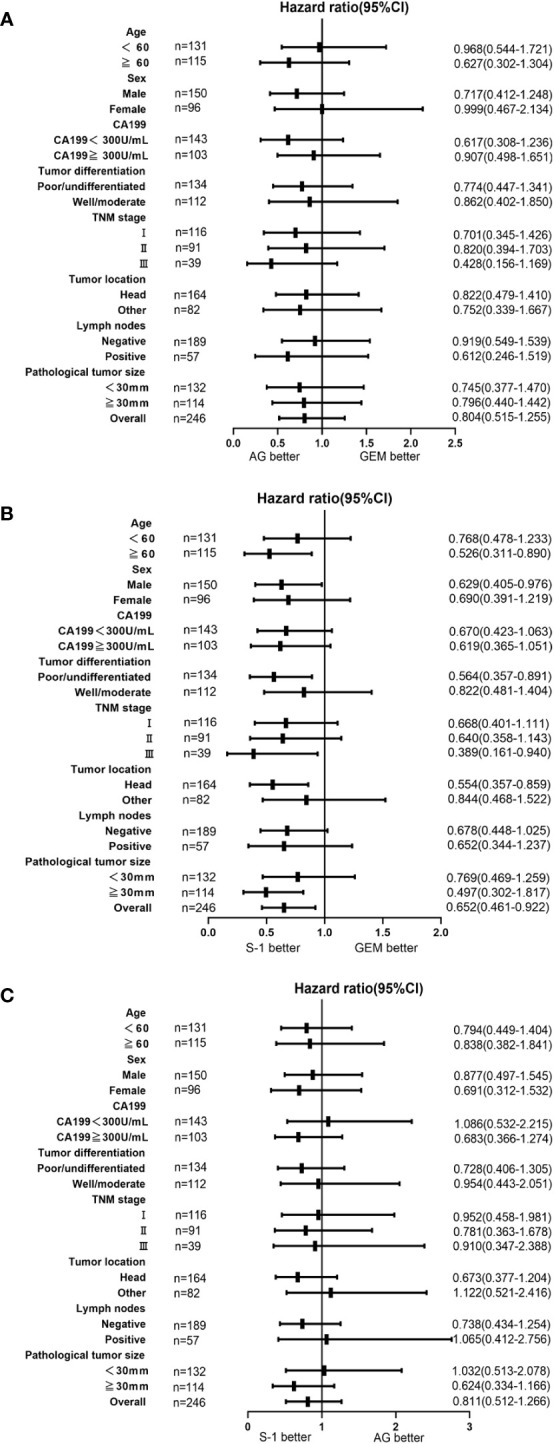
Forest plot of the treatment effect on overall survival in subgroup analysis. Forest plot of the treatment effect on overall survival in prespecified subgroups. **(A)**Comparison between AG and GEM. **(B)** Comparison between S-1 and GEM. **(C)** Comparison between S-1 and AG.

Subgroup analyses demonstrated comparable or superior survival with adjuvant AG or S-1 chemotherapy compared to GEM chemotherapy in almost all subgroups (**Figures 4A, B**). As for the comparison between S-1 chemotherapy and AG chemotherapy, patients who received S-1 chemotherapy had comparable or superior survival to patients who received AG chemotherapy in most subgroups, while for patients with CA19-9 value<300, lymph nodes positive, or tumor size <30mm, AG chemotherapy seemed better for the survival (**Figure 4C**). Significant differences between S-1 chemotherapy and AG chemotherapy were not observed for most subgroups.

## 4 Discussion

In this retrospective real-world study, we compared adjuvant AG and S-1 with adjuvant GEM in patients with PDAC in daily clinical practice. Adjuvant chemotherapy with S-1 was related to a prolonged OS compared to GEM monotherapy (median OS S-1 vs GEM: 27.0 vs 20.0 months; HR: 0.65, 95% CI 0.46-0.82, P = .016) and a significantly prolonged RFS compared to GEM monotherapy (median RFS S-1 vs GEM: 20.0 vs 8.2 months; HR: 0.58, 95% CI 0.41-0.82, P = .002). After adjusting for known prognostic factors in multivariate Cox regression analysis, this survival benefit persists and is consistent in most subgroups in our subgroup analysis. However, no statistically significant differences in OS or RFS were seen between the patients treated with AG and patients treated with GEM or between the patients treated with AG and patients treated with S-1.

In 2018, clinical trials conducted by Conroy et al. found that the median OS of patients receiving adjuvant-modified FOLFIRINOX (fluorouracil, leucovorin, irinotecan and oxaliplatin) prolonged compared to GEM (median OS: 54.0 vs 35.5 months HR: 0.64,95% CI 0.48-86, P =.003) (13), which proves the survival advantage of patients treated with modified FOLFIRINOX (mFOLFIRINOX). However, since this obvious survival advantage is at the expense of increased chemotherapy-related adverse events, international guidelines recommend the use of adjuvant mFOLFIRINOX only in patients with good performance status (9, 18–20). In the clinical practice of our center, AG, S-1, and GEM chemotherapy schemes have been used more frequently as a result of higher safety and lower adverse event rates since the relevant clinical trial results were published (12, 16).

The APACT study in 2019 showed that the AG chemotherapy prolonged overall survival and investigator-assessed disease-free survival, while did not prolong independently-assessed disease-free survival compared with gemcitabine monotherapy after radical resection of pancreatic cancer (16) (median OS AG vs GEM: 41.8 vs 37.7 months; HR 0.82; 95% Cl, 0.687 - 0.973; P= 0.0232; investigator-assessed median DFS AG vs GEM: 16.6 vs 13.7 months; HR 0.82; 95% Cl, 0.694 - 0.965; P = 0.0168; independently-assessed median DFS AG vs GEM: 19.4 vs 18.8 months; HR: 0.88; 95% Cl, 0.729 - 1.063; P = 0.1824). Since its main research endpoint (independently-assessed median DFS) did not achieve positive results, the international guidelines have not recommended its use as a postoperative adjuvant therapy for pancreatic cancer (18–20). In China, the AG scheme for postoperative adjuvant chemotherapy in patients with pancreatic cancer has been written into CSCO (CHINESE SOCIETY OF CLINICAL ONCOLOGY) guidelines as a grade II recommendation since the relevant clinical trial results were published. However, no statistically significant differences in OS or RFS were seen between the patients treated with AG and patients treated with GEM in this study (median OS AG vs GEM: 22.0 vs 20.0 months; HR: 0.80, 95% CI 0.52-1.26, P = .336; median RFS AG vs GEM: 16.0 vs 8.2 months; HR: 0.70, 95% CI 0.45-1.09, P = .113). This is in contrast with the APACT study in 2019. One possible explanation for this difference is that our patients do not accept randomization and there is a risk of subsequent confusion of indications. Although our study showed no difference in baseline characteristics between AG and GEM, the possible effects of increased residual confounding factors cannot be completely excluded (21). Another possible reason is that the sample size included in our study is not large enough, resulting in the difference between the two treatment groups not being statistically presented. Nevertheless, the results of clinical trials are not always reproducible in the real world. Although the AG scheme as a chemotherapy scheme for unresectable pancreatic cancer or borderline resectable pancreatic cancer has been widely recognized (22–26), the extent to which AG chemotherapy as an adjuvant chemotherapy scheme can benefit resectable pancreatic cancer patients after surgery remains to be further studied.

In 2016, a clinical trial conducted in Japan showed that S-1 was superior to gemcitabine as adjuvant therapy (12). The median overall survival was 25.5 months (95% CI 22.5-29.6) with gemcitabine and 46.5 months (37.8-63.7) with S-1. The HR for mortality of S-1, compared with gemcitabine, was 0.57 (95% CI 0.44-0.72, P<0.0001). The median RFS was 11.3 months (95% CI 9.7-13.6) in the gemcitabine group and 22.9 months (17.4-30.6) in the S-1 group. The HR for relapse of S-1, compared with gemcitabine, was 0.60 (95% CI 0.47-0.76, P<0.0001). Therefore, the S-1 scheme has long been the preferred scheme for adjuvant chemotherapy after pancreatic cancer surgery in Japan (27, 28). However, all patients enrolled in this Japanese clinical trial were East Asian residents of Japan. It is reported that there may be differences in pharmacokinetics and pharmacodynamics of S-1 between European and North American patients and East Asian patients. Grade 3 or 4 gastrointestinal toxicity (especially diarrhea) is more common in European and North American patients than in East Asian patients (29, 30), so that it has not yet been recommended as postoperative adjuvant chemotherapy for pancreatic cancer by the international guidelines (18, 20). However, our clinical practice demonstrates that the S-1 scheme does provide survival benefits for patients with resectable pancreatic cancer in the real world, which corresponds to the positive effect in the JASPAC01 trial.

Besides, the median OS of patients treated with GEM in this study (20.0 months) was lower than that of the APACT trial in 2019 (36.2 months) (16) and the JASPAC01 trial in 2016 (25.5 months) (12), which may be attributed to stricter selection criteria in randomized clinical trials, including only patients with good physical status (ECOG PS 0 or 1), serum carbohydrate antigen (CA) 19-9 levels below 100U/mL. However, the patient’s physical condition, preoperative CA19-9 value, and the TNM staging of the tumor were not required according to the design of our study.

This is the first study to compare the adjuvant AG scheme, S-1 scheme and adjuvant GEM scheme in the treatment of resectable PDAC in real-world daily clinical practice. However, our research also has some limitations. First, the number of patients included in this study is not enough, and it is a single-center study, which leads to relatively poor universality of the results. Besides, some data inherent in the retrospective study design are incomplete, which is solved by multiple interpolations in multivariate Cox regression analysis. Furthermore, as the international guidelines do not recommend postoperative adjuvant chemotherapy for people who have received neoadjuvant chemotherapy (20), patients receiving neoadjuvant therapy are excluded from our study, thus limiting the universality of this specific population. Finally, although we adjusted for many variables, not all possible prognostic variables were available, as the analysis results have residual confounding risks. More in-depth studies are urgently needed to assess which of these contemporary multiple chemotherapy schemes shows the most favorable results.

## 5 Conclusion

To conclude, this real-world retrospective study demonstrated that S-1 chemotherapy is associated with better OS and RFS as compared to GEM chemotherapy, while the survival advantage of AG chemotherapy is not statistically significant compared to GEM. As a result, adjuvant S-1 should be preferred over GEM in patients after surgical resection. Whether the AG scheme as an adjuvant chemotherapy scheme can benefit resectable pancreatic cancer patients after surgery remains to be further studied.

## Data availability statement

The raw data supporting the conclusions of this article will be made available by the authors, without undue reservation.

## Ethics statement

The studies involving humans were approved by Ethics Committee of Tianjin Medical University Cancer Institute and Hospital. The studies were conducted in accordance with the local legislation and institutional requirements. Written informed consent for participation in this study was provided by the participants’ legal guardians/next of kin.

## Author contributions

HL: Data curation, Writing – original draft, Writing – review & editing. YG: Writing – original draft. XS: Writing – original draft. YL: Writing – original draft. SC: Writing – original draft. XW: Writing – review & editing. SG: Writing – review & editing. CG: Writing – review & editing. TZ: Supervision, Writing – review & editing.
